# Cognitive impairment in patients with clinically isolated syndrome

**DOI:** 10.1590/S1980-57642012DN06040011

**Published:** 2012

**Authors:** Carolina Fiorin Anhoque, Laurentino Biccas Neto, Simone Cristina Aires Domingues, Antônio Lúcio Teixeira, Renan Barros Domingues

**Affiliations:** 1 Neuroscience Postgraduate Program, Federal University of Minas Gerais, Belo Horizonte MG, Brazil.; 2 Ophthalmology Professor, Surgery Department, Santa Casa School of Health Sciences, Vitória ES, Brazil.; 3 Neuropsychologist, Vitória ES, Brazil.; 4 Professor of Neurology, Neuropsychiatric Branch, Neurology Unit, University Hospital and Neuroscience Postgraduate Program, Federal University of Minas Gerais, Belo Horizonte MG, Brazil.; 5 Professor, Department of Pathology and Internal Medicine, Santa Casa School of Health Sciences, Vitória ES, Brazil and Neuroscience Postgraduate Program, Federal University of Minas Gerais, Belo Horizonte MG, Brazil.

**Keywords:** cognition, clinically isolated syndrome, neuropsychological tests

## Abstract

Cognitive abnormalities have been extensively studied in Multiple Sclerosis (MS). However, little is known about the cognitive involvement in patients with Clinically Isolated Syndrome (CIS).

**Objective:**

This study aimed to investigate cognitive impairment in patients with CIS compared with healthy subjects.

**Methods:**

18 CIS patients and 18 controls were subjected to the Wechsler memory scale, Rey Auditory Verbal Learning, Rey Complex Figure, Paced Auditory Serial Addition, Digit Span, verbal fluency, Stroop color card test, D2, and Digit Symbol tests.

**Results:**

CIS patients had significantly worse performance on the Paced Auditory Serial Addition Test (PASAT) 2 seconds (P=0.009) and on verbal fluency tests (P=0.0038) than controls.

**Conclusion:**

CIS patients had worse cognitive performance than controls on neuropsychological tests evaluating executive functioning.

## Abstract (pt)

As alterações cognitivas na Esclerose Múltipla (EM) têm sido bastante estudadas. No entanto, ainda são poucos os estudos acerca do comprometimento cognitivo em pacientes com Síndrome Clinicamente Isolada (SCI).

**Objetivo:**

O objetivo deste estudo foi o de investigar funções cognitivas em pacientes com SCI em relação a um grupo controle.

**Métodos:**

Dezoito pacientes com SCI e 18 controles saudáveis foram submetidos à avaliação neuropsicológica, incluindo os seguintes testes: Escala Wechsler de Memória, "Rey Auditory Verbal Learning Test", Figura Complexa de Rey, "Paced Auditory Serial Addition (PASAT) 2 e 3 segundos, "Digit Span", fluência verbal, teste de Stroop, D2 e "Digit Symbol Test".

**Resultados:**

Pacientes com SCI tiveram desempenho significativamente inferior nos testes PASAT 2 segundos (P=0.009) e fluência verbal (P=0.0038) quando comparados ao grupo controle.

**Conclusão:**

Pacientes com síndrome clinicamente isolada apresentaram pior desempenho cognitivo em testes relacionados a funções executivas.

## INTRODUCTION

Clinically Isolated Syndrome (CIS) is defined as the first episode of a demyelinating and inflammatory disease of the central nervous system (CNS). A number of patients with CIS will convert to Multiple Sclerosis (MS), a chronic demyelinating disorder characterized by CNS lesions disseminated over time and space.^1^

Recent studies have demonstrated that cognitive dysfunction has a negative impact on the quality of life of such MS patients.^2,3^ Consequently, the study of cognitive function in MS has gained great importance. Cognitive dysfunction is found in 40 to 65% of MS patients^4^ and seems to be related with the number and localization of demyelinating lesions, axonal loss, and brain atrophy typically found in MS.^5^ Considering that these pathological features are progressive, it is important to establish in which phase of the disease the cognitive dysfunction begins. In this regard, some studies assessing cognitive functions in early MS and also in patients with CIS have been conducted.^3,4,6-9^. Several recent studies have shown that CIS patients may present mild cognitive impairment, especially in executive functions.^7,8,10^

The aim of the present study was to investigate a series of cognitive domains in Brazilian patients with CIS compared to healthy subjects.

## METHODS

**Subjects.** Subjects aged 19-48 with CIS were recruited over a two-year period from the Multiple Sclerosis Clinic of the Santa Casa School of Health Sciences, Vitória, Espírito Santo - Brazil. The control group was composed of healthy subjects paired by age, gender, and educational level. The study was approved by the Research Ethics Committee of the Federal University of Minas Gerais, Belo Horizonte, and Santa Casa School of Health Sciences, Vitória, Brazil, and informed consent was obtained from each participant. The diagnosis of CIS was defined according to the following criteria: one isolated neurological episode lasting at least 24 hours compatible with demyelination of the CNS and magnetic resonance imaging showing at least two lesions resembling those seen in MS.^1^ Patients with the first de-myelinating episode with gadolinium-enhancing and non-enhancing lesions on baseline magnetic resonance imaging (MRI) were excluded according to the recent MS diagnostic criteria.^12^ Patients with severe cognitive impairment, defined as a score below 24 points on the Mini-Mental State Examination,^11^ or using psychotropic drugs were not included. All patients had not been using corticosteroids for at least three months leading up to the time of evaluation.

**Neurologic and neuropsychological evaluation.** The clinical evaluation included neurologic examination and determination of current disability using the *Expanded Disability Status Scale* (EDSS)^13^.

CIS subjects and control individuals were subjected to neuropsychological evaluation comprising: verbal learning (Rey Auditory Verbal Learning Test); verbal memory (logical memory subtest from the Wechsler memory scale-revised); constructional ability and visual memory (Rey Complex Figure); and attention and executive function tests: speed of information processing, sustained and divided attention (Paced Auditory Serial Addition Test 3 and 2 seconds); working memory (Digit Span Test from the Wechsler memory scale revised), category restricted verbal fluency (letter and animals); selective attention and cognitive flexibility (Stroop Color test); concentration (D2 test); visual scanning, tracking, and motoric speed (Digit Symbol Test). The cognitive evaluation was performed in an air-conditioned environment at the same temperature.

**Statistical analysis.** Analyses were performed using 'R' software, version 2.8.0. The normality of data distribution was assessed with the Shapiro Wilk test. As data presented a non-normal distribution, the Mann-Whitney test was used to compare neuropsychological parameters between CIS patients and controls. The level of significance was set at p<0.05.

## RESULTS

The mean±SD time between demyelinating episode and cognitive assessment was 17.7±18.2 months. Demographic and clinical data of patients and controls are shown in Table 1. Eighteen CIS patients were included, 13 female and 5 male. The mean±SD age was 35.5±9.1 years. The mean±SD EDSS score of patients with CIS was 0.8±0.5. The mean±SD MEEM score and Wechsler Adult Intelligence Scale (WAIS) of patients with CIS was 28.4±1.3and 113.3±10.1and control group was 29.1±0.9and 115.0±9.1, respectively.

**Table 1:** Demographic and clinical data of patients with clinically isolated syndrome (CIS) and controls.

	Groups	N	Mean (±SD)	p-value
Age	Patients	18	35.6 (9.3)	
	Controls	18	35.5 (9.2)	
Gender	Female	13		
	Male	5		
EDSS	Patients	18	0.8 (0.5)	
Education (years)	Patients	18	14.1 (4.3)	
	Controls	18	14.2 (4.2)	
MMSE	Patients	18	28.4 (1.3)	0.18
	Controls	18	29.1 (0.9)	
Wechsler Adult Intelligence Scale (WAIS)	Patients	18	113.3 (10.1)	0.36
	Controls	18	115.0 (9.1)	
CIS symptom localization	Lobar	2		
	Brainstem	1		
	Spinal cord	5		
	Optic neuritis	10		

SD: standard deviation.

In Table 2, the median scores on each neuropsycho-logical test of patients and controls are shown. The performance of CIS patients in PASAT 2 seconds (correct answer), PASAT 2 seconds (no responses) and Fluency (with letter "S") was significantly lower than controls.

**Table 2:** Cognitive results of patients with clinically isolated syndrome (CIS) and controls.

	Median		P-value
	**Patients**	**Controls**	
Logical Memory (Immediate Recall)	11	15	0.692
Logical Memory (Delayed Recall)	11.5	12	0.924
PASAT 3 Seconds (Correct responses)	40	54.5	0.222
PASAT 3 Seconds (False responses)	2.5	1	0.220
PASAT 3 Seconds (Absence)	14	4	0.188
PASAT 2 Seconds (Correct responses)	18	46.5	0.0216*
PASAT 2 Seconds (False responses)	2	2.5	0.721
PASAT 2 Seconds (Absence)	41	9.5	0.009*
Fluency (Letter)	12.5	15	0.0038*
Fluency (Animals)	19	20.5	0.899
STROOP (Time) (Card3)	22.5	20	0.366
RALVT (Immediate Recall)	9.5	10	0.975
RALVT (Delayed Recall 30')	10	10	0.898
RALVT (Total)	69	73	0.751
Rey Figure (Copy)	36	36	0.771
Rey Figure (Delayed 3')	21	20	0.691
Digit Symbol (Score)	61.5	62.5	0.837
Digit Span (Forward)	6.5	7.5	0.185
Digit Span (Backward)	5	6	0.109
Digit Span (Total)	11.5	14	0.127
D2 (Gross)	446	434.5	0.805
D2 (Total Error)	15	14	0.754
D2 (Net)	434	413	0.717
D2 (Amplitude Oscillation)	12	12	0.716

* Significant.

## DISCUSSION

To our knowledge, this was the first study evaluating cognitive functions in a series of Brazilian CIS patients. Previous studies have shown differences in cognitive performance between CIS patients and controls. Reduced semantic verbal fluency and delayed spatial recall^7^, reduced information processing speed,^3,15-17^ changes in executive function,^3^ abnormalities in working and verbal memory^6^ have been shown in CIS patients. Our results are in line with previous studies showing executive dysfunction in CIS.^7,8,14,15,17^ Specifically, we found abnormalities in speed of information processing, sustained and divided attention, and category restricted verbal fluency; however, there was no difference in other executive functions such as working memory, selective attention and cognitive flexibility, concentration, and visual scanning, tracking, and motoric speed. Therefore, the present study confirmed that CIS may lead to mild cognitive impairment with variable executive dysfunction. This may have clinical implications. Given CIS is the first clinical manifestation of MS and that cognitive abnormalities can be found in CIS, our data suggest that neuropsy-chological evaluation as well neuropsychological follow-up should be carried out soon after the first clinical episode of demyelination. This neuropsychological follow-up will allow a better understanding of the progression of cognitive findings in CIS and in MS.

The present study has some clear limitations. The sample size may be considered small and consequently may have led to underestimation of the differences between groups. Another limitation is the lack of systematic neu-roimaging evaluation, precluding the determination of any correlation between cognitive findings and neuroimaging parameters. Future studies should evaluate the correlation between cognition, topography of MRI lesions and lesion load in patients with CIS. We were not able to evaluate the impact of psychiatric comorbidities such as depression and anxiety on cognition and did not evaluate fatigue symptoms or their impact on cognition. Future and larger studies with multivariate analysis should be able to assess the potential interference of psychiatric syndromes on cognitive test performance in CIS patients.

In conclusion, this study confirmed that mild executive dysfunction occurs in CIS. Future studies are needed to address whether this cognitive compromise is long lasting and/or associated with significant impact on daily activities.
